# Effect of Machine Learning on Dispatcher Recognition of Out-of-Hospital Cardiac Arrest During Calls to Emergency Medical Services: A Randomized Clinical Trial

**DOI:** 10.1001/jamanetworkopen.2020.32320

**Published:** 2021-01-06

**Authors:** Stig Nikolaj Blomberg, Helle Collatz Christensen, Freddy Lippert, Annette Kjær Ersbøll, Christian Torp-Petersen, Michael R. Sayre, Peter J. Kudenchuk, Fredrik Folke

**Affiliations:** 1 Copenhagen Emergency Medical Services, Copenhagen, Denmark; 2 Department of Clinical Medicine, Faculty of Health and Medical Sciences, University of Copenhagen, Copenhagen, Denmark; 3 Danish Clinical Quality Program, National Clinical Registries, Denmark; 4 National Institute of Public Health, University of Southern Denmark, Copenhagen, Denmark; 5 Department of Clinical Investigation, Nordsjællands Hospital, Hillrød, Denmark; 6 Department of Emergency Medicine, University of Washington, Seattle; 7 Department of Medicine, University of Washington, Seattle; 8 Department of Cardiology, Herlev Gentofte University Hospital, Copenhagen, Denmark

## Abstract

**Importance:**

Emergency medical dispatchers fail to identify approximately 25% of cases of out-of-hospital cardiac arrest (OHCA), resulting in lost opportunities to save lives by initiating cardiopulmonary resuscitation.

**Objective:**

To examine how a machine learning model trained to identify OHCA and alert dispatchers during emergency calls affected OHCA recognition and response.

**Design, Setting, and Participants:**

This double-masked, 2-group, randomized clinical trial analyzed all calls to emergency number 112 (equivalent to 911) in Denmark. Calls were processed by a machine learning model using speech recognition software. The machine learning model assessed ongoing calls, and calls in which the model identified OHCA were randomized. The trial was performed at Copenhagen Emergency Medical Services, Denmark, between September 1, 2018, and December 31, 2019.

**Intervention:**

Dispatchers in the intervention group were alerted when the machine learning model identified out-of-hospital cardiac arrest, and those in the control group followed normal protocols without alert.

**Main Outcomes and Measures:**

The primary end point was the rate of dispatcher recognition of subsequently confirmed OHCA.

**Results:**

A total of 169 049 emergency calls were examined, of which the machine learning model identified 5242 as suspected OHCA. Calls were randomized to control (2661 [50.8%]) or intervention (2581 [49.2%]) groups. Of these, 336 (12.6%) and 318 (12.3%), respectively, had confirmed OHCA. The mean (SD) age among of these 654 patients was 70 (16.1) years, and 419 of 627 patients (67.8%) with known gender were men. Dispatchers in the intervention group recognized 296 confirmed OHCA cases (93.1%) with machine learning assistance compared with 304 confirmed OHCA cases (90.5%) using standard protocols without machine learning assistance (*P* = .15). Machine learning alerts alone had a significantly higher sensitivity than dispatchers without alerts for confirmed OHCA (85.0% vs 77.5%; *P* < .001) but lower specificity (97.4% vs 99.6%; *P* < .001) and positive predictive value (17.8% vs 55.8%; *P* < .001).

**Conclusions and Relevance:**

This randomized clinical trial did not find any significant improvement in dispatchers’ ability to recognize cardiac arrest when supported by machine learning even though artificial intelligence did surpass human recognition.

**Trial Registration:**

ClinicalTrials.gov Identifier: NCT04219306

## Key Points

**Question:**

Can a machine learning model help medical dispatchers improve recognition of out-of-hospital cardiac arrest?

**Findings:**

In this randomized clinical trial of 5242 emergency calls, a machine learning model listening to calls could alert the medical dispatchers in cases of suspected cardiac arrest. There was no significant improvement in recognition of out-of-hospital cardiac arrest during calls on which the model alerted dispatchers vs those on which it did not; however, the machine learning model had higher sensitivity that dispatchers alone.

**Meaning:**

These findings suggest that while a machine learning model recognized a significantly greater number of out-of-hospital cardiac arrests than dispatchers alone, this did not translate into improved cardiac arrest recognition by dispatchers.

## Teaser

This randomized clinical trial examines how a machine learning model trained to identify out-of-hospital cardiac arrest and alert dispatchers during emergency calls affected the recognition of and response to out-of-hospital cardiac arrests.

## Introduction

Survival after out-of-hospital cardiac arrest (OHCA) has increased in several countries following improvements in bystander interventions, the response of emergency medical services (EMS), and postresuscitation care. Of these, early bystander interventions, particularly cardiopulmonary resuscitation (CPR) with early defibrillation, can have the greatest potential impact on outcome.^1^ Rapid recognition of cardiac arrest by the emergency medical dispatcher followed by instructing the caller to promptly perform CPR and retrieve an automated external defibrillator are essential steps.^2,3,4,5^ Such guidance is contingent on prompt recognition of OHCA by the dispatcher. In 2018, the overall rate of CPR by bystanders was 77% in Denmark,^6^ which corresponded to the rate of dispatcher-recognized OHCA at the Copenhagen Emergency Medical Services (EMS).^3,7^

A rate-limiting step to initiating bystander CPR and expediting EMS response lies in the recognition of OHCA by dispatchers. A promising strategy for improving OHCA recognition is the use of artificial intelligence systems based on deep neural networks to provide real-time information to the dispatcher. Such systems estimate the likelihood of OHCA based on the patterns of the words spoken that might be missed by the dispatcher.

A clinical decision support tool based on machine learning models was tested at the Copenhagen EMS and was able to identify OHCA with better sensitivity and only slightly lower specificity than medical dispatchers.^7^ Despite the potential for such clinical decision support tools to improve health care outcomes by offering data-driven insights, almost all decision support tools driven by artificial intelligence or machine learning have so far failed to do so in practice.^8^ Thus, while the translation of research techniques like machine learning into clinical practice presents a new frontier of opportunity, the real-world deployment of this modality remains rare and in need of further exploration.^9^

In this randomized clinical trial, we investigated the effect of real-time information on dispatchers’ ability to recognize OHCA during emergency calls. Our primary aim was to examine whether the machine learning model affected the clinical practice of medical dispatchers. We hypothesized that the machine learning model would increase recognition of OHCA when dispatchers were augmented with machine learning compared with standard call procedures. Secondary outcomes were differences between the 2 approaches in time-to-recognition of OHCA, initiation of dispatcher-assisted CPR (DA-CPR), and time to its initiation.

## Methods

### Ethical Approval

We followed the General Data Protection Regulation and registered the study at the Danish Data Protection Agency. The study is approved by the Danish Patient Safety Authority. The research ethics committee in the Capital Region of Denmark waived the need for ethical approval. The study followed the Consolidated Standards of Reporting Trials (CONSORT) reporting guideline. The regional ethical committee waived the need for preregistration of this study. However, we retrospectively registered this trial with ClinicalTrials.gov in August 2019. We obtained written informed consent from all study participants, ie, medical dispatchers.

### Setting

The study was performed in Copenhagen, Denmark, with a population of 1.8 million persons and a size of 2563 km^2^. Copenhagen EMS handles approximately 130 000 emergency calls annually. The emergency phone number, 112, connects to the Emergency Medical Dispatch Centre, which is staffed by medically trained dispatchers comprised of nurses (70%) and paramedics (30%) who receive 6 weeks of training in communication, prioritization of emergency calls, and DA-CPR instructions. In cases of suspected OHCA, dispatchers instruct callers in CPR while simultaneously dispatching an ambulance and a physician-manned Mobile Critical Care Unit.

### Emergency Call Processing

Emergency calls to Copenhagen EMS are analyzed by the previously described machine learning model,^7^ which listens to every call and can immediately alert the dispatcher when the model suspects OHCA. The machine learning model in this study is identical to that used previously^7^ and estimates OHCA with a 1-second resolution, meaning that for each second of conversation between caller and dispatcher, the machine learning model calculates the probability of whether there is an OHCA in the accumulated call information. If the probability of the machine learning model exceeds a prespecified threshold, the call is defined as a suspected OHCA and a warning can be issued to the dispatcher.

### Trial Design

This double-masked, 2-group, randomized clinical trial (RCT) evaluated a machine learning model that analyzes calls in real time and delivers decision support to medically trained dispatchers compared with conventional call-handling without decision support from the machine learning model. A more extensive description of the study is available in the trial protocol in Supplement 1.

#### Eligibility and Randomization

All emergency calls received between September 1, 2018, and December 31, 2019, were considered for trial enrollment. However, only calls that the machine learning model suspected as OHCA were eligible for randomization to the intervention or control group. Calls placed on hold while the dispatcher conferred with EMS physicians were excluded if the machine learning alert only appeared after the hold. In addition, calls were excluded according to predefined postrandomization exclusions^10^: if the machine learning model erroneously identified an OHCA that was not subsequently confirmed by the Danish Cardiac Arrest Registry; calls with a broken chain of alert (eg, calls that were forwarded from out-of-hours nonemergency services); repeated calls on the same incident; calls from police requesting help from the EMS; and calls regarding EMS-witnessed OHCAs. Finally, calls for which CPR had been initiated prior to the call were excluded as an already confirmed OHCA.

Calls meeting the inclusion criteria were assigned to either the intervention or control group in a 1:1 ratio, using a random number generating program with no stratification factors.^11,12^ Medical dispatchers were masked to their group assignment. Unless the machine learning model generated and disclosed an OHCA alert, neither group knew whether the machine learning model was operative during the call. Investigators involved in data analysis and outcome assessment were also masked to group assignment.

In a post hoc analysis, we examined compliance to the machine learning model and the consequences if the intervention group had heeded every machine learning alert. We compared the time of the machine learning alert in the intervention group with the dispatcher’s time of OHCA recognition (without such an alert) in the control group.

#### Participant Procedures

All calls to EMS Copenhagen are transferred from the police and start with a conference between the police and medical dispatcher. In case of OHCAs that are recognized by the dispatcher, time to recognition is measured from the start of the call, which includes the conference time. After recognition of OHCA, the next event in the call is initiation of DA-CPR.

All dispatchers participated in the trial and were instructed to follow a specified protocol when receiving an alert from the machine learning model.^13^ When the alert appeared, the dispatcher had the option to ignore the alert, or if heeded, they were instructed to immediately dispatch an ambulance and a physician-staffed vehicle on the suspicion of OHCA. The precise time of the machine learning alert, both those heeded and ignored, was recorded and stored in a central database. The dispatcher’s reaction to the alert, whether heeded or ignored, did not influence the enrollment or subsequent analysis. For the control group, the alerts generated by the machine learning model were suppressed and not shown to the dispatchers. However, the time when the machine learning alert occurred, although not directed to the dispatcher, was recorded. The alert was designed as a conspicuous display on the dispatcher’s main screen while not otherwise interfering in the dispatcher’s work.

#### Case Review

All emergency dispatch calls were recorded. To verify confirmed OHCA, calls were linked to the Danish Cardiac Arrest Registry. Calls for which the machine learning model had identified a true OHCA were analyzed by a group of trained evaluators who were masked to the randomization assignment using a modified dispatch data survey catalog from the American Cardiac Arrest Registry to Enhance Survival.^14,15^ Case review allowed us to collect data for the secondary end points, ie, time to recognition and time to DA-CPR.

#### Outcomes

The primary outcome was the rate of dispatchers’ recognition of subsequently confirmed OHCA. Secondary outcomes were dispatchers’ time to recognition of OHCA and the rate of DA-CPR, defined as when the dispatcher instructed the caller to place hands on the patient’s chest or similar action directives.

### Statistical Analysis

The performance of the dispatchers in the control and intervention groups was compared using the Danish Cardiac Arrest Registry as the reference for a confirmed OHCA as was the overall performance of the machine learning model. Results are reported with 95% CIs and *P* values when appropriate. Time-to-event analyses were conducted with Kaplan-Meier failure curves to estimate time to recognition in each group and for the machine leaning model overall.

In assessing the machine learning performance, calls classified by the model as OHCA and confirmed by the registry reference standard were considered true-positive findings. The Mann-Whitney test was used to compare calls classified as OHCA and non-OHCA by the machine learning model. A 2-tailed *P* < .05 was considered significant for all analyses. Data management and statistical analyses were performed using SAS version 9.4 (SAS Institute).

Based on our previous study,^7^ the difference in recognition between machine learning model and dispatcher was 10%. Setting the significance level (α) at 5% and the power (1 − β) at 95%, 356 calls were needed in each group, resulting in a total study sample of 712 calls.

## Results

**Figure 1.**
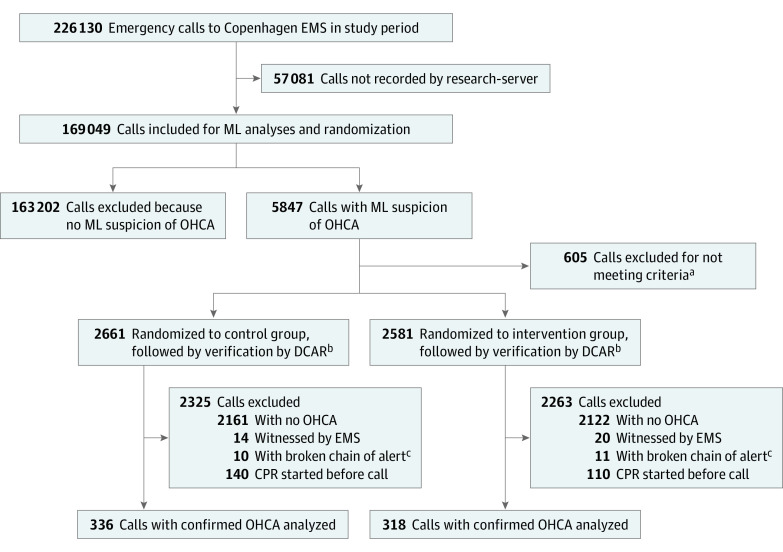
Study Flowchart DCAR indicates Danish Cardiac Arrest Registry; EMS, emergency medical services; ML, machine learning; OHCA, out-of-hospital cardiac arrest. ^a^Calls that had been on hold while dispatcher conferred with EMS physicians did not meet inclusion criteria if the alert appeared after the conference. ^b^All calls were linked to the DCAR by the personal identification number. If a call had a corresponding cardiac arrest in the register, it was included in further analysis. ^c^Calls forwarded from out-of-hours services or calls from other authorities requesting help were excluded.

During the intervention period, Copenhagen EMS received 226 130 calls, while the machine learning model processed only 169 049 calls (74.7%) due to downtime. The downtime was randomly spread across the day and across dispatchers, showing equal distribution between OHCA calls and non-OHCA calls and not affecting the randomization. Of the 169 049 calls processed by the machine learning model during the 16-month study period, the machine learning model identified 5847 calls as suspected OHCA. Of these, 5242 (89.7%) were eligible for randomization and were randomized to either control (2661 [50.8%]) or intervention (2581 [49.2%]) dispatcher groups; 4588 (87.5%) of the calls were excluded after randomization (Figure 1).

The characteristics of the included patients are shown in Table 1. A total of 654 patients were enrolled, of whom 318 (48.6%) were randomly assigned to the intervention group and 336 (51.4%) to the control group. The mean (SD) age was 70 (16.1) years across both groups, 419 of 627 patients (67.8%) with known gender were men, and 332 (50.8%) had OHCAs witnessed by bystanders; most OHCAs (539 [82.4%]) took place in a residential setting. Caller and patient characteristics were similar between groups.

**Table 1.** Baseline Characteristics

Characteristic	Calls, No. (%)	
	Control group (n = 336)	Intervention (n = 318)
Patient age, mean (SD), y	69.1 (16.1)	71.4 (15.6)
Male patients^a^	217 (67.8)	202 (65.8)
Call length, mean (SD), min	6.68 (3.39)	6.93 (3.36)
Conference time, mean (SD), s	15.1 (7.5)	14.6 (9.0)
OHCA location		
Residential	268 (79.8)	271 (85.2)
Public	33 (9.8)	28 (8.8)
Other	63 (18.8)	42 (13.2)
OHCA witnessed	178 (53.1)	154 (48.4)
Shockable rhythm	71 (22.1)	62 (20.3)
Male caller	111 (33.1)	96 (30.3)
Caller alone at time of call	146 (43.6)	154 (48.6)
Caller relationship to patient		
Health care	76 (22.6)	89 (28.0)
Relative	183 (54.5)	162 (50.9)
Other	77 (22.9)	67 (21.1)
Caller access to patient		
By patient’s side	301 (89.6)	283 (89.0)
Not by patient’s side but can access the patient	24 (7.1)	17 (5.4)
Cannot access the patient	8 (2.4)	17 (5.4)
Defibrillated by EMS	87 (26.0)	81 (25.5)
ROSC	97 (28.9)	101 (31.8)

Abbreviations: EMS, emergency medical services; OHCA, out-of-hospital cardiac arrest; ROSC, return of spontaneous circulation.

^a^ Data on gender were missing for 27 patients.

### Primary and Secondary Outcomes

**Figure 2.**
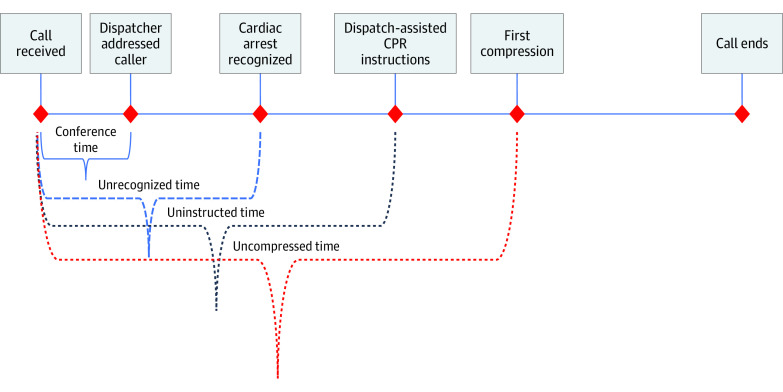
Timeline for Call to 112 CPR indicates cardiopulmonary resuscitation.

In the analysis of primary outcomes, 654 calls were eligible for analysis. From the randomized calls with verified OHCA, the medical dispatchers recognized 296 of 318 (93.1%) in the intervention group and 304 of 336 (90.5%) in the control group, with no significant difference between the 2 groups (*P* = .15). In the 304 calls in the intervention group, the mean (SD) time to dispatchers OHCA recognition was 1.72 (1.52) minutes vs 1.70 (1.63) minutes in 296 calls in the control group (*P* = .90) (Figure 2).

In all 318 calls in the intervention group, the machine learning model recognized the OHCA and generated an alert in a mean (SD) of 1.39 (1.32) minutes from the emergency call’s onset, a difference of only 0.05 minutes compared with the suppressed alert not displayed in the 336 calls in the control group (mean [SD] time, 1.33 [1.51] minutes; *P* = .60)

Dispatchers started CPR instructions in 206 calls (64.8%) in the intervention group and 208 calls (61.9%) in the control group (*P* = .47), with no significant difference in elapsed time from when the OHCA was recognized. Elapsed time without CPR instructions was 2 seconds longer in the intervention than control group (Table 2).

**Table 2.** Primary and Secondary Outcomes

Outcome	Group, mean (SD)		*P* value
	Control	Intervention	
Eligible for analysis, No. (%)	336 (51.5)	318 (48.5)	.48
Call length, min	6.68 (3.39)	6.94 (3.36)	.35
Alert generated from machine learning model, min^a^	1.33 (1.51)	1.39 (1.32)	.60
Recognition of cardiac arrest, No (%)	304 (90.5)	296 (93.7)	.15
Secondary outcomes			
Time to dispatcher recognition, min	1.70 (1.57)	1.71 (1.63)	.90
DA-CPR instructions started, No. (%)	208 (61.9)	206 (64.8)	.47
Time to DA-CPR, min	2.48 (1.89)	2.52 (1.76)	.82

Abbreviation: DA-CPR, dispatcher-assisted cardiopulmonary resuscitation.

^a^ Alert shown in intervention group only.

In the intervention group, dispatchers’ reaction time from receiving the alert to their recognition of an OHCA was 20 seconds. The corresponding interval was 22 seconds from when the suppressed alert might have first appeared to the control group.

### Machine Learning Model Compliance

**Figure 3.**
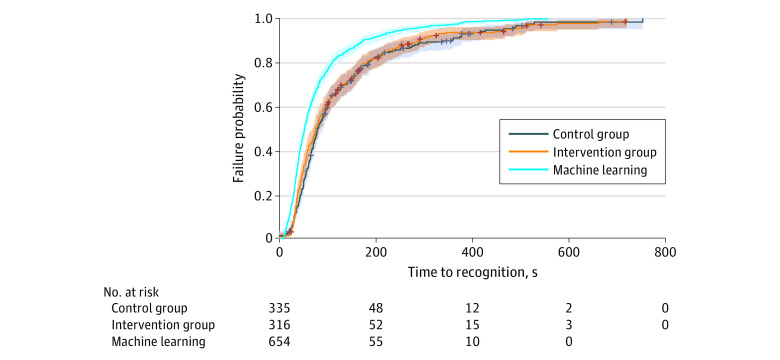
Failure Curves for Recognition of Out-of-Hospital Cardiac Arrest The shaded areas indicate 95% CIs; crosses, censored calls, ie, those that ended without the dispatcher recognizing the condition.

Figure 3 shows how the machine learning model continually outperformed the dispatchers, both in the intervention group and in the control group. In the intervention group, 22 additional OHCAs would have been recognized by the dispatcher had they heeded the alert, and the 296 OHCAs would have been recognized 20 seconds sooner than they were in the absence of such an alert (mean [SD] time, 1.38 [1.32] minutes vs 1.71 [1.63] minutes; *P* = .008).

### Overall Accuracy of the Machine Learning Model

We examined the 169 049 calls processed by the machine learning model, of which 1110 (0.7%) were OHCAs unwitnessed by EMS and confirmed by the Danish Cardiac Registry. Compared with medical dispatchers, the machine learning model had a significantly higher sensitivity (77.5% vs 85.0%; *P* < .001) but lower specificity (99.6% vs 97.4%; *P* < .001). The machine learning model had a significant lower positive predictive value than dispatchers (17.8% vs 55.8%; *P* < .001). Put in pragmatic terms, if the dispatchers had heeded the machine learning model alert, 54 additional OHCAs that were not recognized by dispatchers would have been recognized.

The machine learning model resulted in a sizeable number of false alerts in which an OHCA had not actually occurred. Thus, complete compliance with both the true and false alerts from the model in the intervention group would have resulted in 2122 cases of a false-positive OHCA (2122 of 2581 [82.2%] of all suspected OHCA calls). Had all such alerts also been disclosed to and heeded by the control group, 2161 cases (81.2%) of false-positive OHCAs would have been produced. From the 4283 false alerts, 1519 (35.5%) received a response other than a full OHCA response, increasing the total number of ambulances dispatched from 56 449 to 57 968, a 2.7% increase.

## Discussion

The current study was designed as an RCT that also addressed the paucity of RCTs investigating artificial intelligence.^16^ To our knowledge, this study is the only published study analyzing machine learning models processing speech in a real-time conversation between a medical dispatcher and patient or bystander, during which the system automatically extracts information from the conversation to be transformed into real-time decision support for the medical professional. There are only a few RCTs testing artificial intelligence and machine learning technologies, all of which either report negative or insignificant findings.^16,17,18^

This study tested a novel approach to telephone triage, in which a machine learning model automatically extracted information in real-time and transformed this into decision support for the medical dispatcher recognizing OHCA. The machine learning model was found to correctly recognize more OHCAs and do this significantly faster than the dispatchers. However, the use of a machine learning model in the current setting did not result in an increased number of correct recognitions of OHCA by dispatchers, and the time to recognition of OHCA was unchanged.

In 169 049 calls to Copenhagen EMS, including 1110 calls regarding OHCA, we found that the machine learning model could correctly recognize OHCA calls in more than 85.0% of OHCA calls, which is similar to a previous retrospective study, in which we found a sensitivity of 84.1%.^7^ The specificity was also similar to the retrospective study, with 97.4% compared with 97.3%. Thus, the developed machine learning decision support tool performs in a live, prospective setting with similar results to the retrospective studies. It is through prospective studies, preferably RCTs, that we can understand the utility of machine learning models, given that performance is likely to be worse when encountering real-world data that differ from those encountered in algorithm training.^9^ The very similar results of the prospective and retrospective studies demonstrates the robustness of the model, validating the basic principles of the machine learning model.

We further examined the consequences of compliance to all alerts from the machine learning model. This would have resulted in 54 additional OHCAs being recognized. Conversely, in a scenario with 100% compliance, 1519 ambulances would have been dispatched as part of a full OHCA response, increasing the total number of dispatches by 2.7%, from 56 449 to 57 968 ambulances dispatched with lights and sirens.

There is a paucity of literature about the clinical performance of machine learning models or artificial intelligence analyzing calls to support medical dispatchers. Experience with machine learning models has been limited to retrospective studies, whereas the current study showed higher sensitivity and less time to decision on suspected OHCA compared with medically trained dispatchers. However, as shown in this trial, being alerted to a suspected OHCA did not significantly affect the behavior of the dispatchers who responded to the OHCA call. The results underpin the need for discussing implementation when adapting new technology. These results are similar to the results of the Computerised Interpretation of Fetal Heart Rate During Labor trial and other RCTs testing machine learning models or artificial intelligence in clinical practice, which found no evidence of computerized decision support reducing the likelihood of poor outcomes.^17,18^

The human factor in the interaction with decision support tools is crucial, and several studies report hesitancy in the interaction with decision support tools.^19,20^ Our findings are concordant with a review focusing on compliance with decision support tools, which reported that actual use rates remain low despite clinicians acknowledging these technologies as useful.^21^

While the machine learning model delivers robust advice with high sensitivity, it appears that dispatchers did not comply with alerts. Further consideration is needed before implementing such a tool in clinical practice to determine whether further training of medical professionals can remedy the missing compliance.

We found that creating a machine learning model with high sensitivity was not sufficient to improve recognition of OHCA. It would also be necessary to establish confidence in the machine learning model. We found that extreme obedience to the machine learning model would have resulted in more OHCAs being recognized and reduced the time to recognition, although with more frequent high-priority dispatch of ambulances.

### Limitations

This study has limitations. A potential weakness is that the medical dispatchers can learn from prior exposure to the machine learning model when not passive and in turn improve outcomes in the control group when encountering an OHCA call during which the model remains passive. In hindsight, we should have provided more education to dispatchers to improve compliance with the machine learning model. Third, the servers analyzing the phone calls had downtime because the server was underdimensioned. To remediate this, the randomization period was prolonged.

## Conclusions

In this RCT, we did not find any significant improvement in dispatcher recognition of OHCA when supported by machine learning, even though artificial intelligence did surpass human recognition. This suggests that machine learning has the potential to positively affect the recognition rate of OHCA while also improving the rate of DA-CPR, but these findings did not lead to improved recognition of OHCA or improved rate of DA-CPR. Future studies are needed to improve human-computer interaction. However, efforts should be made to improve the specificity of the machine learning model to improve the relevance of alerts.

## Notes

Supplement 1. **Trial Protocol**

Supplement 2. **Data Sharing Statement**
