# Supplementary material for: Effect of Machine Learning on Dispatcher Recognition of Out-of-Hospital Cardiac Arrest During Calls to Emergency Medical Services: A Randomized Clinical Trial
Source: JAMA Netw Open. 2021 Jan 6;4(1):e2032320. doi: 10.1001/jamanetworkopen.2020.32320 (PMC7788469; doi:10.1001/jamanetworkopen.2020.32320)
Supplement: Supplement 1. — Trial Protocol [file jamanetwopen-e2032320-s001.pdf]

Randomized clinical trial of machine learning assisted recognition of Out-of-Hospital Cardiac Arrest during emergency calls versus non-assisted recognition of Out-of-Hospital Cardiac Arrest during emergency calls. The study protocol.

Indhold

- Background 2
- Aim 3
- Methodology 4
  - Participants 4
  - Flow 4
  - Intervention 5
  - Primary outcome measure 5
  - Secondary outcome measures 5
  - Sample size 6
  - Randomization 6
  - Blinding 6
  - Statistical analysis plan 6
  - Timeline and ethics 7
- List of abbreviations 7
- Project organisation 7
- Funding 8
- Acknowledgements 8

Background

Survival from OHCA depends on four links in the chain of survival: Recognition of the event, CPR, defibrillation and post resuscitation care[1,2].

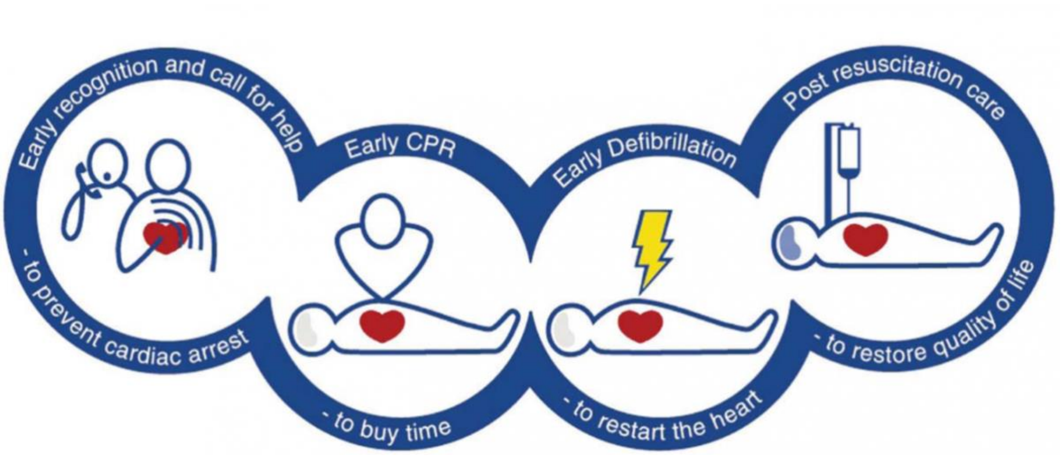

Early recognition and intervention are critical for patient survival. High quality CPR and defibrillation by AED prior to EMS arrival improves survival after OHCA[3-5]. Therefore, the chance of surviving OHCA is highly correlated with bystander and medical dispatchers' recognition of the condition during the emergency calls.

Previous studies have investigated patterns in medical dispatchers' recognition of OHCA[6-10].

However, still not all OHCAs are recognised when incorporating these patterns in the algorithms used by medical dispatchers[11]. In two recent studies, the recognition rate of OHCA in EMDC Copenhagen was established to be 70 % and 81 %[12,13]. While this is considered a high accuracy, it still leaves room for improvement.

Bystanders recognise about 30 % of OHCA before calling the emergency number. The remaining OHCA are recognised during the interview with the medical dispatcher[13]. Medical dispatchers often have special training in identifying cardiac arrest, however, OHCA constitutes only few percentages of the total call volume. The individual medical dispatcher thereby gets little experience in recognising OHCA. To support the medical dispatcher in decision-making, most dispatch centre have included algorisms in their dispatch system to guide the medical dispatcher. Some dispatch centres supply with systematic ongoing feedback as quality assurance. However, the combination of human experiences and use of algorithm does not nearly identify all cardiac arrests.

Although improvements have been made during the past few decades, survival after OHCA remains low[3,14-16]. The period immediately after OHCA is critical; each instance that passes in which the patient does not receive resuscitation, greatly decreases their chance of survival[17,18]. The time from collapse to EMS arrival is often more than 5 minutes; this delay, coupled with the need for immediate resuscitative support, emphasises the critical importance of having early interventions performed by bystanders with guided assistance from medical dispatchers.

At the EMDC-Copenhagen, the medical dispatcher will by the use of 'Danish Index', and basic algorithms "No-No-Go" usually recognise OHCA. The "No-No-Go" approach is a potential tool for improving OHCA recognition. This method is a two-question approach, where the medical dispatcher in every emergency call asks the caller if the patient is conscious and if the patient is breathing normally. If the answer for both questions is "No", then relevant EMS units are dispatched, and medical dispatcher-assisted CPR protocols are initiated ("Go"). A novel approach to improve recognition of OHCA is to apply machine learning directly to the dialogue.

In a previous project "Can a computer through machine learning recognise of Out-of-Hospital Cardiac Arrest during emergency calls" (supported by TrygFoundation), we found, it was possible to create a Machine Learning model, which could recognise OHCA with higher precision than medical dispatchers at the EMDC-Copenhagen.

We now wish to test and document the effect of the model in the EMDC-Copenhagen. For this purpose, we have built a server running the ML-model. This server is integrated in the network at EMDC-Copenhagen, making it possible to push alerts to the medical dispatcher, when a cardiac arrest is recognised by the model.

With aid of machine learning, the hypothesis is, that recognition of OHCA is improved, and happen both more frequent and faster than present.

An instruction for the medical dispatchers is developed, which guides the medical dispatcher in instance of an alert from the machine.

Aim

1. To investigate whether a potential increase in recognitions is due to machine alerts or the increased focus of the medical dispatcher on recognizing OHCA when implementing the machine
2. To investigate if a machine learning model based on neural networks, when alerting medical dispatchers will increase overall recognition of OHCA and increase dispatch of citizen responders.

3. To investigate increased use of AEDs, CPR or dispatch of citizen responders in cases of OHCA on machine recognised OHCA vs. medical dispatcher recognised OHCA.

Methodology

The study has been designed as a prospective, blinded, randomized clinical trial (RCT). Each call where the machine learning model suspects a cardiac arrest is by lot (1:1) randomized to either alert on dispatchers' screen or no alert on dispatchers' screen.

This trial will comply with the CONSORT 2010 Statement[19] and the SPIRIT guidelines[20] and a Statistical Analysis Plan (SAP)[21] will be included.

Participants

800 participants/calls will be included consecutively in the study, from EMS-Copenhagen covering all the capital region.

Inclusion/exclusion criteria are listed in Table 1.

Table 1

Inclusion and Exclusion Criteria

Inclusion Criteria	Exclusion Criteria:
- Call regarding a cardiac arrest registered in the national Danish Cardiac Arrest Registry<br>- OHCA is recognized by machine-learning model<br>- Call originates from 1-1-2	- OHCA EMS-witnessed<br>- Call is from another authority (police or fire brigade)<br>- Call is a repeat call<br>- Call has been on hold for conference

Flow

All calls to 1-1-2 EMS-Copenhagen are analysed by the machine-learning model. When the model recognizes a suspected OHCA, lot is drawn (1:1), and an alert will be shown on screen for the intervention group. For the control-group call is answered following usual guidelines and routines.

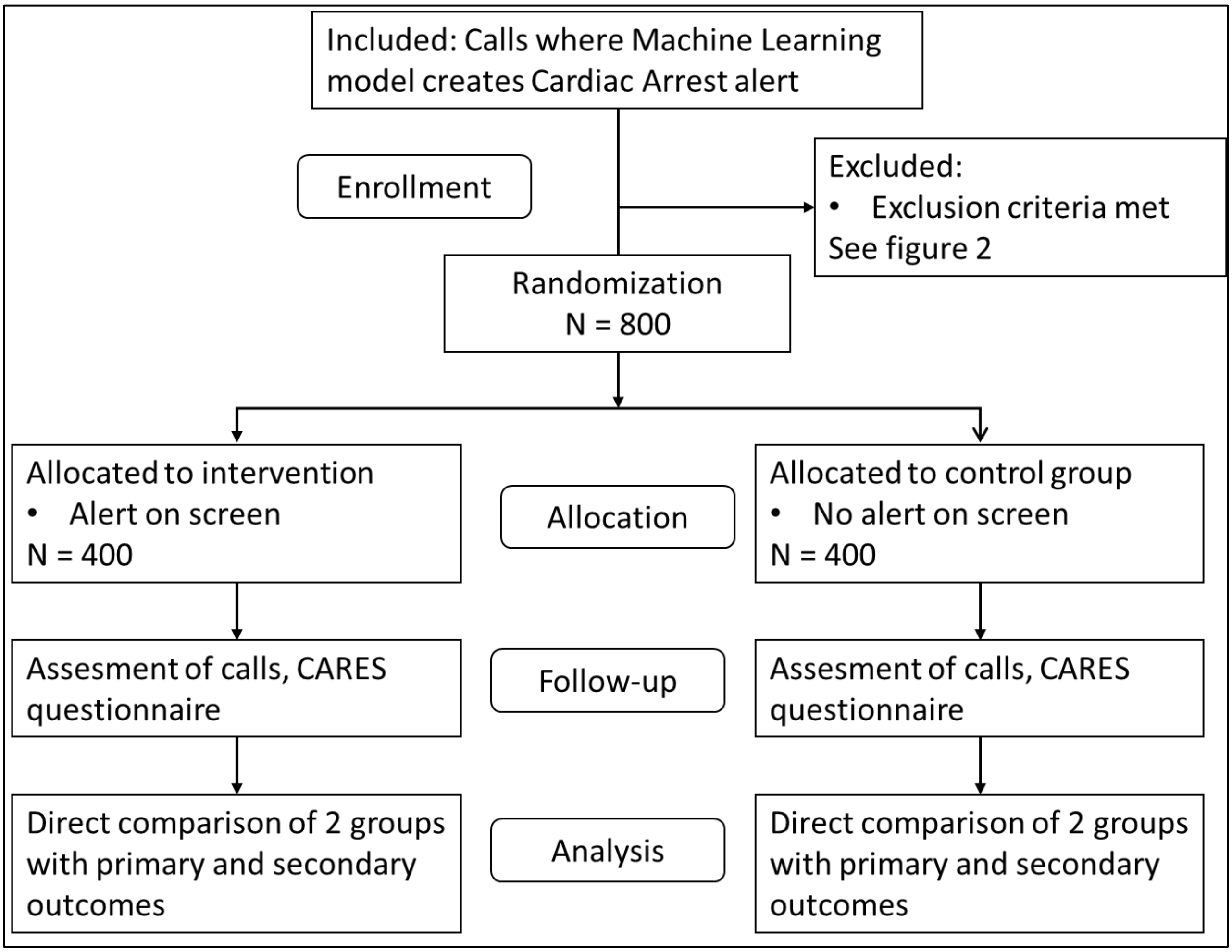

Fig. 2

Intervention

Based on the randomization of the patient/call, the dispatcher/call taker will either have 1) alert on the screen, 2) no help from the machine learning model, and the dispatcher will proceed along the usual guidelines.

All dispatchers from EMS-Copenhagen will participate, and all dispatchers are medically trained call takers, and either trained as paramedics or nurse (RN).

Primary outcome measure

Dispatcher recognition of out-of-hospital cardiac arrest is the primary outcome. Recognition is reported by a questionnaire filled in by a group of auditors listening to recordings of all included calls. The questionnaire is a modified CARES protocol for XXX calls and consists of 21 questions whereby the quality of the call is evaluated. The questionnaire is validated and has been used in other studies.

Secondary outcome measures

Secondary outcomes measures are derived from the CARES-question are.

Time to recognition

Dispatcher assisted CPR initiated

Time of CPR / first compression

Sample size

Based on our previous study[22], the difference in recognition between machine learning model and dispatcher is 10 %. Setting the significance level (alpha) at 5 % and the power (1-beta) at 95 %, 356 calls is needed in each group, resulting in a total study population of 712 calls. This number has been rounded up to 800 by the research-group.

Randomization

Simple randomization with a 1:1 allocation between intervention and control group. Randomization is done at time of (machine) recognition of cardiac arrest.

The random allocation sequence is made digitally by the machine learning model picking a random number between 0 and 1, allocating to the two groups depending on value being more or less than 0.5.

Blinding

Randomization is blinded to dispatchers, who will not know whether a call is in the control-group.

Statistical analysis plan

Following analysis are performed at the end of the trial for both intervention and control group

- All OHCA are identified with data from the Prehospital Patient Journal to confirm whether there was an actual OHCA
- Was OHCA recognized by medical dispatcher
- Was Cardiopulmonary resuscitation (CPR) initiated
- How many seconds before OHCA is recognised
- Follow up on whether the medical dispatcher followed the alert
- How did medical dispatchers with alerts react upon false alerts?
- Was the alert correct

Analysis is done by comparing

- Recognition of OHCA
- Time of CPR initiation
- Time (seconds) before OHCA is recognized
- Follow up on whether the medical dispatcher followed the alert
- How did medical dispatchers with alerts react upon false alerts?

Analyse will cover

1. Median recognition time – machine compared to medical dispatchers
2. Percentage of OHCA recognised – machine compared to medical dispatcher
3. Characterise machine recognition
    a. False positives
    b. False negatives
4. Characterise medical dispatcher recognition
    a. False positives
    b. False negatives

Timeline and ethics

Recruitment of calls/patients started September 2018. Based on expected incidence of OHCA, inclusion is expected to last between 9 and 11 months as a conservative estimate.

The trial protocol has been approved by the Regional Ethics Committee of the Capital region of Denmark.

List of abbreviations

AED — Automated external defibrillator

CPR — Cardiopulmonary resuscitation

DCAR — Danish Cardiac Arrest Registry

EMDC — Emergency medical dispatch centre

EMS — Emergency Medical Services

MCCU —Mobile Critical Care Unit

OHCA — Out-of-Hospital cardiac arrest

ROSC — Return of spontaneous circulation

Project organisation

Primary investigator

Stig Nikolaj Blomberg, MSc., PhD-fellow, Specialist Consultant, Emergency Medical Services, Copenhagen, The Capital Region of Denmark

In this project, the primary investigator is responsible for data collection, data analysis, first-drafts of papers and publications. In the first year of the study, he has validated data for the algorithm, planned and

executed the entire project, and made interim analysis. Prior to this, he has through his work in EMS Copenhagen extensive knowledge on the mechanisms and processes in a call to EMDC-Copenhagen. He has years' experience as data manager, and an extensive knowledge on the data structure of the EMDC. He also has statistical knowledge with respect to both methods and statistics in prehospital research.

The Co-investigators are from different universities and contribute with interdisciplinary skills. The combined group of skills within the project covers project leadership, emergency medicine, anaesthesiology, register-based research, register-based epidemiology, statistics,

Co-investigators:

Freddy K. Lippert, M.D. Associate professor, The Faculty of Health and Medical Sciences, Department of Clinical Medicine, University of Copenhagen, Denmark. CEO, Emergency Medical Services, Copenhagen, The Capital Region of Denmark. Contribute with guidance and review.

Helle Collatz Christensen, M.D. Ph.D., Emergency Medical Services, Copenhagen, The Capital Region of Denmark. Contribute with guidance and review.

Frederik Folke, M.D. Ph.D., Associate professor, Emergency Medical Services, Copenhagen and the Faculty of Health and Medical Sciences, Department of Clinical Medicine, University of Copenhagen, Denmark. Contribute with guidance, methodological and clinical counselling and review.

Annette Kjær Ersbøll, MSc, Professor, PhD, National Institute of Public Health, University of Southern Denmark. Contribute with guidance, statistical analysis and review.

Funding

This study is supported by an unrestricted grant from the Danish foundation TrygFonden and The Laerdal Foundation.

Acknowledgements

We thank Corti.ai who created the machine learning framework.

Reference List

1. Cummins RO, Ornato JP, Thies WH, Pepe PE. Improving survival from sudden cardiac arrest: the "chain of survival" concept. A statement for health professionals from the Advanced Cardiac Life Support Subcommittee and the Emergency Cardiac Care Committee, American Heart Association. Circulation 1991;83:1832-47.
2. Perkins GD, Handley AJ, Koster RW, et al. European Resuscitation Council Guidelines for Resuscitation 2015: Section 2. Adult basic life support and automated external defibrillation. Resuscitation 2015;95:81-99.
3. Wissenberg M, Lippert FK, Folke F, et al. Association of national initiatives to improve cardiac arrest management with rates of bystander intervention and patient survival after out-of-hospital cardiac arrest. JAMA 2013;310:1377-84.
4. Stiell IG, Brown SP, Nichol G, et al. What is the optimal chest compression depth during out-of-hospital cardiac arrest resuscitation of adult patients? Circulation 2014;130:1962-70.
5. Blom MT, Beesems SG, Homma PC, et al. Improved survival after out-of-hospital cardiac arrest and use of automated external defibrillators. Circulation 2014;130:1868-75.
6. Roppolo LP, Westfall A, Pepe PE, et al. Dispatcher assessments for agonal breathing improve detection of cardiac arrest. Resuscitation 2009;80:769-72.
7. Bohm K, Stalhandske B, Rosenqvist M, Ulfvarson J, Hollenberg J, Svensson L. Tuition of emergency medical dispatchers in the recognition of agonal respiration increases the use of telephone assisted CPR. Resuscitation 2009;80:1025-8.
8. Bohm K, Vaillancourt C, Charette ML, Dunford J, Castren M. In patients with out-of-hospital cardiac arrest, does the provision of dispatch cardiopulmonary resuscitation instructions as opposed to no instructions improve outcome: a systematic review of the literature. Resuscitation 2011;82:1490-5.
9. Bang A, Herlitz J, Martinell S. Interaction between emergency medical dispatcher and caller in suspected out-of-hospital cardiac arrest calls with focus on agonal breathing. A review of 100 tape recordings of true cardiac arrest cases. Resuscitation 2003;56:25-34.
10. Breckwoldt J, Schloesser S, Arntz HR. Perceptions of collapse and assessment of cardiac arrest by bystanders of out-of-hospital cardiac arrest (OOHCA). Resuscitation 2009;80:1108-13.
11. Alfsen D, Moller TP, Egerod I, Lippert FK. Barriers to recognition of out-of-hospital cardiac arrest during emergency medical calls: a qualitative inductive thematic analysis. Scand J Trauma Resusc Emerg Med 2015;23:70.
12. Moller TP, Andrell C, Viereck S, Todorova L, Friberg H, Lippert FK. Recognition of out-of-hospital cardiac arrest by medical dispatchers in emergency medical dispatch centres in two countries. Resuscitation 2016;109:1-8.
13. Viereck S, Møller TP, Ersbøll AK, et al. Recognising out-of-hospital cardiac arrest during emergency calls increases bystander cardiopulmonary resuscitation and survival. Resuscitation 2017;115:141-7.
14. Sasson C, Rogers MA, Dahl J, Kellermann AL. Predictors of survival from out-of-hospital cardiac arrest: a systematic review and meta-analysis. Circ Cardiovasc Qual Outcomes 2010;3:63-81.
15. Grasner JT, Lefering R, Koster RW, et al. EuReCa ONE-27 Nations, ONE Europe, ONE Registry: A prospective one month analysis of out-of-hospital cardiac arrest outcomes in 27 countries in Europe. Resuscitation 2016;105:188-95.
16. Stromsoe A, Svensson L, Axelsson AB, et al. Improved outcome in Sweden after out-of-hospital cardiac arrest and possible association with improvements in every link in the chain of survival. Eur Heart J 2015;36:863-71.
17. Larsen MP, Eisenberg MS, Cummins RO, Hallstrom AP. Predicting survival from out-of-hospital cardiac arrest: a graphic model. Ann Emerg Med 1993;22:1652-8.
18. Holmberg M, Holmberg S, Herlitz J. Incidence, duration and survival of ventricular fibrillation in out-of-hospital cardiac arrest patients in sweden. Resuscitation 2000;44:7-17.
19. Schulz KF, Altman DG, Moher D. CONSORT 2010 Statement: updated guidelines for reporting parallel group randomised trials. BMJ 2010;340:c332.

20. Chan A-W, Tetzlaff JM, Altman DG, et al. SPIRIT 2013 Statement: Defining Standard Protocol Items for Clinical Trials. Annals of Internal Medicine 2013;158:200-7.
21. Gamble C, Krishan A, Stocken D, et al. Guidelines for the Content of Statistical Analysis Plans in Clinical TrialsGuidelines for the Content of Statistical Analysis Plans in Clinical TrialsGuidelines for the Content of Statistical Analysis Plans in Clinical Trials. JAMA 2017;318:2337-43.
22. Blomberg SN, Folke F, Ersbøll AK, et al. Machine learning as a supportive tool to recognize cardiac arrest in emergency calls. Resuscitation 2019.
