# Supplementary material for: Effect of Machine Learning on Dispatcher Recognition of Out-of-Hospital Cardiac Arrest During Calls to Emergency Medical Services: A Randomized Clinical Trial
Source: JAMA Netw Open. 2021 Jan 6;4(1):e2032320. doi: 10.1001/jamanetworkopen.2020.32320 (PMC7788469; doi:10.1001/jamanetworkopen.2020.32320)
Supplement: Supplement 2. — Data Sharing Statement [file jamanetwopen-e2032320-s002.pdf]

Data Sharing Statement

Blomberg. Effect of Machine Learning on Dispatcher Recognition of Out-of-Hospital Cardiac Arrest During Calls to Emergency Medical Services. *JAMA Netw Open*. Published January 06, 2021. doi:10.1001/jamanetworkopen.2020.32320

Data
Data available: Yes
Data types: Deidentified participant data
How to access data: The datasets generated during and/or analysed during the current study are available from the corresponding author on reasonable request by mail to stig.nikolaj.fasmer.blomberg@regionh.dk
When available: With publication

Supporting Documents
Document types: None

Additional Information
Who can access the data: The datasets generated during and/or analysed during the current study are available from the corresponding author on reasonable request by mail to stig.nikolaj.fasmer.blomberg@regionh.dk
Types of analyses: research
Mechanisms of data availability: after approval of a proposal
